# Understanding the basis of space closure in Orthodontics for a more efficient orthodontic treatment

**DOI:** 10.1590/2177-6709.21.2.115-125.sar

**Published:** 2016

**Authors:** Gerson Luiz Ulema Ribeiro, Helder B. Jacob

**Affiliations:** 1 Professor, Universidade Federal de Santa Catarina (UFSC), Undergraduate and Graduate Programs, Department of Orthodontics, Florianópolis, Santa Catarina, Brazil.; 2 Professor, Texas A&M University, Baylor College of Dentistry, Undergraduate and Graduate Programs, Department of Orthodontics, Dallas, Texas, USA.

**Keywords:** Space closure, Biomechanical, Anchorage

## Abstract

**Introduction:**

Space closure is one of the most challenging processes in Orthodontics and requires a solid comprehension of biomechanics in order to avoid undesirable side effects. Understanding the biomechanical basis of space closure better enables clinicians to determine anchorage and treatment options. In spite of the variety of appliance designs, space closure can be performed by means of friction or frictionless mechanics, and each technique has its advantages and disadvantages. Friction mechanics or sliding mechanics is attractive because of its simplicity; the space site is closed by means of elastics or coil springs to provide force, and the brackets slide on the orthodontic archwire. On the other hand, frictionless mechanics uses loop bends to generate force to close the space site, allowing differential moments in the active and reactive units, leading to a less or more anchorage control, depending on the situation.

**Objective:**

This article will discuss various theoretical aspects and methods of space closure based on biomechanical concepts.

## RESUMO

**Introdução:**

O fechamento de espaços é um dos processos mais desafiadores na Ortodontia e requer uma compreensão sólida de conceitos biomecânicos, a fim de se evitar efeitos colaterais indesejáveis. Compreender o fundamento biomecânico do fechamento de espaços possibilita uma melhor definição das opções de ancoragem e tratamento, por parte dos clínicos. Apesar de haver uma variedade de desenhos de aparelhos ortodônticos, o fechamento de espaços pode ser realizado por meio da mecânica com atrito ou sem atrito, e cada técnica apresenta vantagens e desvantagens. A mecânica com atrito, ou mecânica de deslizamento, é atraente em virtude de sua facilidade, o espaço é fechado por meio do uso de elásticos ou molas helicoidais, que produzem força, fazendo com que os braquetes deslizem no arco ortodôntico. Por outro lado, a mecânica sem atrito se utiliza de dobras em alças para gerar força para fechar o espaço, possibilitando momentos diferenciais nas unidades ativa e reativa, induzindo a uma ancoragem mais ou menos controlada, dependendo da situação.

**Objetivo:**

o presente artigo discutirá vários aspectos teóricos e métodos de fechamento de espaços, baseando-se em conceitos biomecânicos.

## INTRODUCTION

Space closure is one of the most challenging processes in Orthodontics. Tooth extraction, molar distalization, expansion of dental arches, interproximal reduction, among other things, have been part of the orthodontic armamentarium to correct malocclusion and allow dental space gain with which the orthodontist should deal. The ability to close spaces, especially those resulting from tooth extraction, is an essential skill required during orthodontic treatment. Space closure mechanics without knowledge can result in failure to achieve an ideal occlusion. Current knowledge in biomechanics, allied with the development of new material and techniques, made significant upgrading possible in space closure, which has simplified mechanics.^1^ ^,^ ^2^ ^,^ ^3^

The biomechanical basis of space closure enables clinicians to determine anchorage and treatment options, reach the prognosis of various alternatives, as well as decide specific adjustments that can improve the outcomes of care. In order to achieve good treatment outcomes, it is crucial to understand the principles behind space closure. Regulation of space closure is ultimately determined by the biomechanical forces applied to the teeth, variation in force and moment magnitude, moment-to-force ratio (M/F), force-to-deflection rate, and anchor unit.^1^

Due to the large number of mechanical options, special attention must be given to the selection of the most appropriate model for each case. Certain aspects must be considered, and precise control of tooth movement during space closure in three dimensions is of preponderant importance to achieve treatment goals. In general, six goals should be considered for space closure: 1) Differential space closure-anchorage control; 2) Minimum patient cooperation; 3) Axial inclination control; 4) Control of rotations and arch width; 5) Optimum biological response; and 6) Operator convenience.

Two basic biomechanical strategies can be used to close spaces: frictionless (closing loop mechanics) and frictional (sliding mechanics). In the early 2010s, 64% of Brazilian orthodontists used the technique based on frictional mechanics, while only 20% of them used more than one technique.^4^ In spite of the variety of appliance designs available to the orthodontist, the techniques of either closing loops or sliding mechanics have their advantages and disadvantages. This article will discuss various theoretical aspects of space closure as well as some methods to close space sites, based on biomechanical concepts.

## ANCHORAGE

Anchorage is something that provides a secure hold. In Orthodontics, it can be defined as the ability to prevent tooth or teeth movement while moving another tooth or group of teeth. In modern Orthodontics, the success of orthodontic treatment generally relies on the anchorage protocol planned for each specific case. Anchorage should be established at the beginning of treatment and its preparation is a very important part of orthodontic treatment.^5^

**Figure 1:**
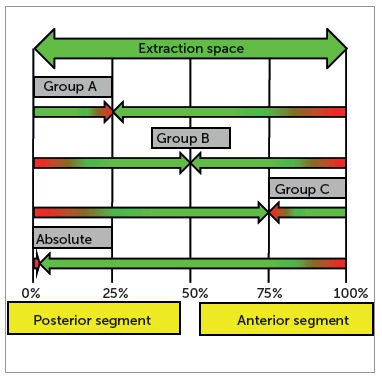
Anchorage classification: Group A space closure includes, on average, 25% of posterior anchorage loss and 75% of anterior retraction; Group B space closure includes more equal amounts of anterior and posterior tooth movement; Group C space closure includes, on average, 75% posterior protraction and 25% of anterior retraction. Absolute anchorage includes practically 100% of anterior retraction.

Depending on the treatment planning, one tooth or group of teeth can be classified as an active unit, while the other is classified as the reactive or passive unit. In general, these two units play different roles during space closure. The active unit is normally affected by the majority of movements, while the other unit resists to movement (anchorage). It is convenient to classify an extraction arch by the differential space closure required between anterior and posterior teeth. One of the most widely used anchorage classification (Fig 1) is applied to the segmented arch technique:^5^ Group A arch is one in which posterior segments must remain in their original position and the full space is used for anterior retraction; Group B arch requires that approximately one half of the space be used for retraction; Group C arch requires that approximately most of the space be closed by protraction of posterior teeth. Nowadays, a fourth type of anchorage can be added to Burstone's classification: absolute anchorage. Clinically, it is very difficult to avoid movement in the passive unit; however, due to skeletal-based anchorage systems, significant steps have been taken towards achieving an absolute anchorage.^1^ ^,^ ^2^ ^,^ ^3^ ^,^ ^5^

Traditionally, orthodontists have developed a variety of strategies and techniques to maintain anchorage.^5-14^ Understanding biomechanical concepts is essential to control anchorage by promoting different types of tooth movement for the active teeth *versus* the reactive unit. From a clinical perspective, delivering appropriate force systems (variation of force, moment magnitude, and moment-to-force ratio) is an important determinant of the resulting tooth movement and maintenance of anchorage.

## BIOMECHANICAL PERSPECTIVE

**Figure 2:**
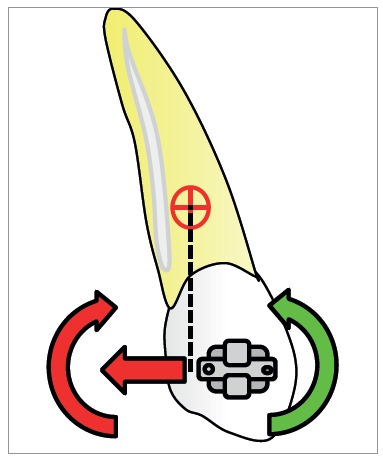
A force that does not pass through the center of resistance produces a rotational movement (moment of force) as well as s linear movement.

**Figure 3:**
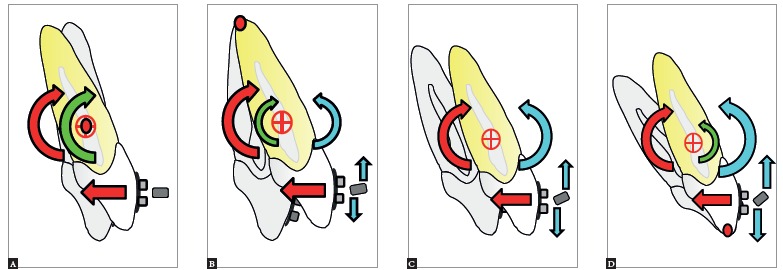
Types of tooth movement: A) Uncontrolled tipping; B) Controlled tipping; C) Bodily movement; D) Root movement. The red arrows represent the force applied to teeth and the moment of force. The blue arrows represent the force of a wire into the bracket and the moment of a couple. The green arrow is the resultant moment (moment of force minus moment of a couple).

The way a tooth moves is dependent on the nature of the force system. The force system includes the force and moments applied to the bracket and the actual force distribution on the periodontium. Force distribution is a function of the tooth's center of rotation.^15^ ^-^ ^22^ By applying a force (F) that does not pass through the center of resistance of the unit to be moved, the orthodontist produces a moment (M_F_) which can cause tipping (Fig 2). The nature of tooth movement can be controlled by applying a counteracting moment (M). The applied M acts in the opposite direction of the M_F_, and moves the root(s) towards the space. As the magnitude of the applied couple increases, the rotation of the tooth would move the crown away from the space. The moment-to-force ratio can determine the quality of tooth movement (Fig 3).^1^ ^-^ ^9^ ^,^ ^13^ ^,^ ^18^ ^,^ ^20^ ^,^ ^22^

**Figure 4:**
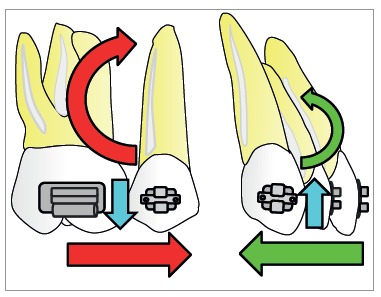
Differential moment reduces the moment/force ratio on one segment while increasing the moment/force ratio on another. Vertical forces occur due to difference in alpha and beta moments.

The retraction force applied by a spring to the active unit is reciprocally applied to the reactive unit. To preserve anchorage, the orthodontist desires greater force for anterior teeth and smaller force for posterior teeth towards the space; external or extra-arch mechanisms must be included (i.e., headgear or miniscrew). Other possibility is differential M/F between active and reactive units. Higher M/F for posterior teeth encourages anchorage preservation, as they resist tipping. Also, the professional should understand that unequal moments between active and reactive units generate vertical forces (Fig 4).^1^ ^,^ ^2^ ^,^ ^3^ ^,^ ^8^ ^,^ ^9^ ^,^ ^10^

## METHODS FOR SPACE CLOSURE

Orthodontic treatment planning is more than just deciding on extraction or nonextraction. Although many approaches towards space closure have been described, the biomechanical principles defining the nature of the force systems applied show many similarities among diverse techniques. Many details determine the tooth movement required during space closure, and it can be performed either by means of frictional or frictionless mechanics.

Applying force by means of coil springs or power chain elastics in sliding mechanics will produce friction between the bracket and the archwire, and the tooth feels less force than the orthodontist is in fact applying. Additionally, the guiding wire provides moments required for prevention of tipping and rotation. In frictionless mechanics, there is no guiding wire, so there is no loss of applied force due to sliding friction. With pros and cons, each technique has its particularities. Simplicity is a goal of clinical practice management, and it may be at odds with the desired biomechanical properties of the appliance.

### Frictional mechanics

Sliding mechanics is attractive due to its simplicity. However, the efficiency of this modality of space closure may be compromised due to friction. Clinically, there are numerous factors that may cause friction. These factors include, among others, bracket slot width, bracket composition, wire size, wire composition, wire-to-slot ligation method, interbracket distance, and relative interface motion between the bracket and the archwire.^23^ ^,^ ^24^ ^,^ ^25^

**Figure 5:**
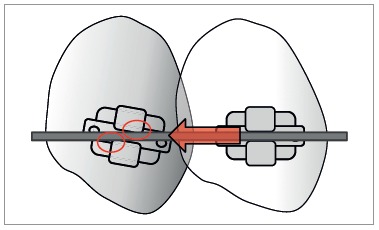
As the canine tips distally during retraction, the orthodontic wire binds against the edge of the bracket slot ("binding effect"), increasing friction.

**Figure 6:**
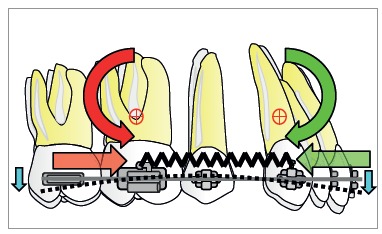
Force system generated by a closed coil spring applying force bellow de center of resistance of the segments. Due to linear distance between the force application and center of resistance, moments occur, and the dumping effect with vertical forces will take part of the space closure.

**Figure 7:**
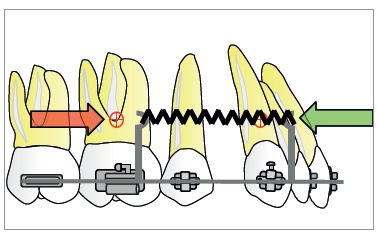
Force system generated by a closed coil spring applying forces at the level of the center of resistance by means of extension hooks (power arms). No moments and vertical forces occur.

Bracket designs and manufacturing techniques have improved to reduce the amount of friction between bracket and wire. Clinical studies support the view that resistance to sliding has little to do with friction; instead, it is largely a binding-and-release phenomenon that does not change considerably with conventional and self-ligating brackets (Fig 5).^26^ As binding delays tooth movement in the active unit, the reactive unit starts to move, causing anchorage loss.^27^ ^,^ ^28^ Accurate control of anterior teeth during space closure in sliding mechanics is essential to the success of orthodontic treatment. When the line of action of force passes below the center of resistance of anterior teeth, a backward moment acts on anterior teeth, resulting in tipping and extrusion of incisors (Fig 6). The orthodontist can add power arms in the anterior segment to provide better vertical control of the anterior segment (Fig 7). When power arms are lengthened, rotation of the entire dentition decreases.^29^ Elastic deformation of the archwire can also be a cause of rotation of anterior teeth.^29^

It is practically impossible for the orthodontist to know the exactly force system due to friction in the sliding mechanics. A small interbracket distance (canine to second premolar, most of the times) does not allow the clinician to apply the differential M/F ratio. Due to the very limited M/F ratio, space closure is normally achieved by group B mechanics. Differential space closure (i.e., group A or group C) may require additional appliances, such as headgear and miniscrews.^29^

### Frictionless mechanics

Orthodontists bend closing loops in a continuous archwire or a segmented arch with a view to delivering forces that can perform space closure. The loops provide the required M/F ratio with great predictability and versatility. Well-designed closing loops promote a more continuous type of movement, and there are many reasons for choosing one configuration over another. Studies on force constancy suggest that continuous forces promote greater rates of tooth displacement.^23^ ^,^ ^26^ ^,^ ^30^

The spring characteristics of the closing loops are mainly determined by some factors, such as wire material, archwire cross-section, interbracket distance, and configuration, and position of the loop. The moment-to-force ratio is probably the most important characteristic of a retraction archwire. Low load/deflection, efficiency and space closure control should be preferred over simplicity of fabrication and delivery.^30^

## LOOP DESIGN

Every orthodontist knows that a wire is stiff, and applying forces at each end will create elongation that is not detectable to the naked eye. The force-deflection rate is too high and would make a useless spring. Adding bends to the wire (i.e., making loops) can dramatically reduce the force-deflection rate. Over the years, different space closure loop configurations have been developed. Some designs have more advantages than others.^30^

Stainless steel tear drop loops are the most common design due to their ease of fabrication; however, they deliver very high forces with only 1 mm of activation.^7^ ^,^ ^27^ Simple loops are associated with small activations and rapid force decay, including intermittent force delivery; thus, having a negative impact on treatment efficiency.^28^ ^,^ ^31^ Also, as shown by Burstone and Koenig,^6^ an error as small as 0.3 mm in the horizontal length of the common vertical loop produces large changes on the M/F ratio, making difference enough to change from root movement to tipping. Due to its characteristics, T-loop has a high M/F ratio and delivers more constant forces over a large deactivation span than vertical loops.^1^

Increasing wire length in the loop design, i.e., adding a helix, or using metal alloys with lower modulus of elasticity (i.e., beta-titanium), reduce the force delivered at the same activation.^27^ Due to the depth of the vestibule, the orthodontist is limited to how high the loop can be made. In order to overcome this problem, a wire, such as a T-loop, can be added horizontally, or there might be addition of helices.

## THE BAUSCHINGER EFFECT

**Figure 8:**
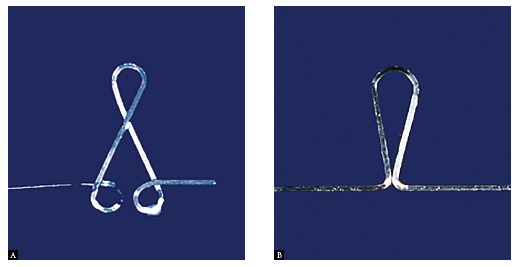
A) Closing loop with bends in the winding-direction. This configuration presents more resistance to permanent deformation during activation; B) Closing loop with bends in unwinding-direction.

The Bauschinger effect is normally associated with conditions in which the strength of a metal decreases when the direction of strain is changed. It is a general phenomenon found in most polycrystalline metals.^32^ In other words, if we have two different T-loop designs, when one closure loop is activated, if all bends are bent in the same direction, it provides more resistance to permanent deformation than if all bends are bent in the opposite direction (Fig 8).

Wire bends should be in the same direction during the processes of forming and activation. Due to some designs, the orthodontist should overbend the wire, following it by a reversal in the direction of the bending, so as to reach the final shape. As a result, the direction of the last bend is correct and provides favorable residual stress during activation. This overbend will provide resistance to permanent deformation, thereby increasing the range of activation. The orthodontist may heat-treat (stress relief) a stainless steel archwire when loop forming does not provide favorable residual stress.

## LOOP POSITION

**Figure 9:**
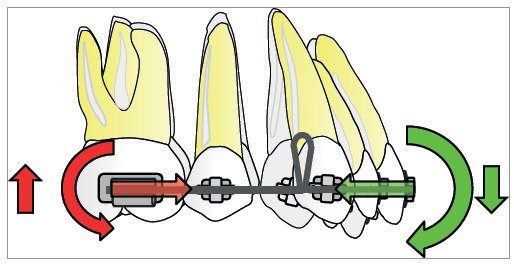
Tear drop loop asymmetrically placed (closer to anterior than posterior segments) provides a very low moment/force ratio with inadequate root control. The advantage of this loop position is the possibility of numerous activations on the same wire as the space closes.

When retracting the anterior segment, the orthodontist normally places closing loops immediately distal to the lateral or canine because this procedure allows for repeated activation of the loop as the space closes. However, it has been shown that the loop position can increase or decrease the amount of posterior anchorage loss.^24^ ^,^ ^31^ If the closing loop is placed off-centered between the anterior and posterior units, the shorter section creates greater moments, encouraging root tipping (increasing anchorage), while the longer section creates smaller moments, encouraging translation.^6^ ^,^ ^15^ ^,^ ^18^ Moreover, asymmetrical placement of the loop between brackets not only results in unequal moments, but also generates vertical forces.^33^ ^,^ ^34^ The vertical forces could lead to a deep overbite relationship. This can be detrimental when a loop is placed closer to anterior teeth due to extrusion (Fig 9).

**Figure 10:**
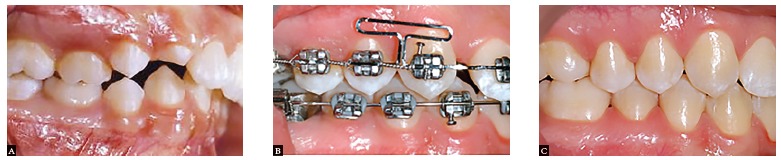
Space closure in a clinical case with non-extraction treatment: A) Initial phase; B) Beginning of the space closure phase; C) End of treatment.

**Figure 11:**
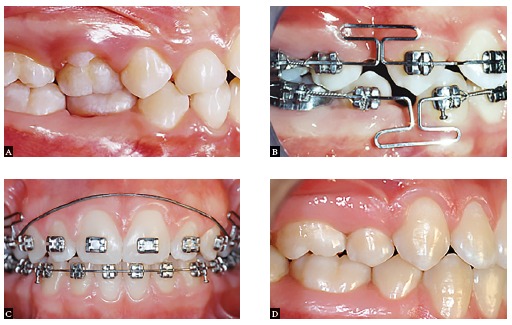
Space closure in a clinical case with extraction treatment: A) Initial phase; B) Beginning of the space closure phase; C) Gable bends: D) End of treatment.

**Figure 12:**
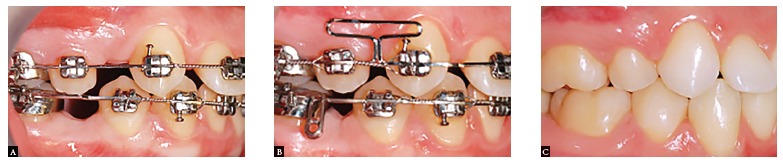
Clinical case with maxillary first premolar extractions and congenitally missing mandibular second premolars. A) End of the alignment and leveling phase; B) Beginning of space closure; C) End of treatment.

**Figure 13:**
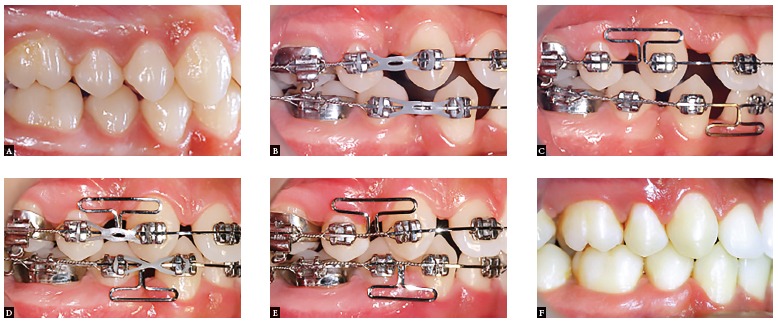
Clinical case with all four first premolar extractions. A) Initial; B) Partial canine retraction; C) Beginning of space closure using a T-loop design; D) Progress of space closure; E) Management of canine relationship; F) End of the case.

**Figure 14:**
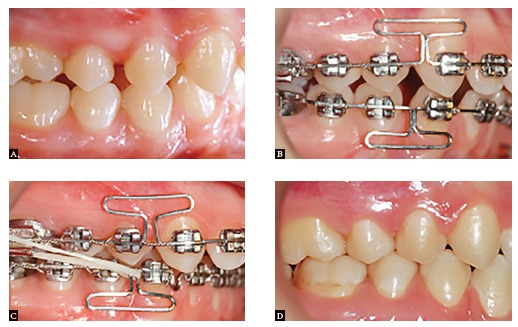
Management of space closure in a surgical case. A) Initial phase; B) Space closure phase; C) Class III elastics to create a differential anchorage control and decompensation of the incisors; D) End of treatment.

**Figure 15:**
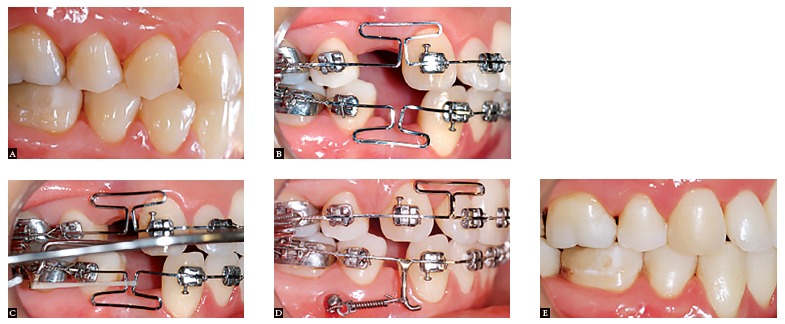
Clinical case with maxillary and mandibular first premolar extractions. A) Initial phase; B) Beginning of space closure; C) Headgear to provide greater anchorage on maxillary molars; D) Frictionless mechanics on maxilla and friction mechanics associated with miniscrew anchorage on the mandible; E) End of treatment.

**Figure 16:**
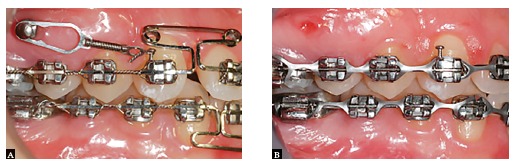
Clinical case without extraction. A) Space closure using miniscrew as anchorage in the maxilla; B) End of the space closure phase.

**Figure 17:**
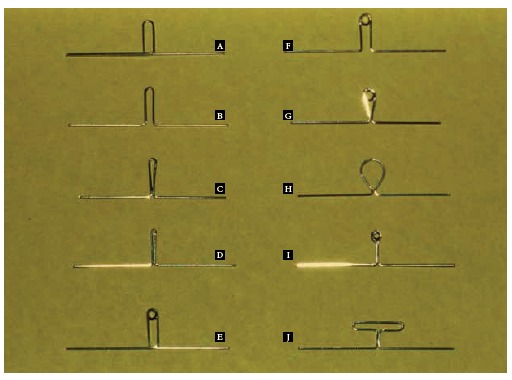
Most common space closure loop designs used by orthodontists: A) reverse vertical loop, B) open vertical loop, C) closed vertical loop, D) bull loop, E) reverse vertical loop with helix, F) open vertical loop with helix, G) closed vertical loop with helix, H) tear drop loop, I) helical loop, J) T-loop.

## ANGLED BENDS AND THE NEUTRAL POSITION 

Orthodontists have learned that, in order to achieve bodily movement, an attraction spring (closing loop) must produce a counter-moment, and the M/F ratio will determine the type of movement (i.e., translation or uncontrolled tipping).^19^ ^-^ ^22^ By ensuring that the ideal force system is produced, orthodontists can place second-order bends (V-bends or gable bends) to increase root control. These preactivation loads are capable of keeping teeth upright during deactivation. Adding gable bends is a common means to adjust the M/F ratio in the anteroposterior direction, thus avoiding dumping of teeth as the space closes.^35^ Gable bends adjust the moment-force ratio to a level that produces the desired unit movement, for the most part of the loop configurations are insufficient to prevent uncontrolled tipping.^5^ ^,^ ^13^ Having a better understanding of the gable effects helps clinicians to achieve desired clinical results, such as increased anchorage control.

The orthodontist needs to understand that gable bends produce angulation, but when the springs are placed only at the occlusal portion, the vertical arms will cross one on top of the other, causing some horizontal force. This will cause more horizontal activation than what is anticipated by the clinician, leading to either permanent deformation or high forces.^34^

The so-called neutral position has no horizontal forces, although some vertical forces may be present. Neutral position is an important concept of a specific shape. The starting position (neutral position) for a zero horizontal force is with vertical arms crossed (when occlusal bends are present). The orthodontist cannot assume that zero force is present, if the vertical arms are just touching.

## ONE-PHASE (EN-MASSE) ***VERSUS*** TWO-PHASE RETRACTION (SINGLE CANINE RETRACTION)

Classically, it has been believed that separated canine retraction followed by four incisors retraction would preserve posterior anchorage. The reason to believe so is because lighter forces could be used at each stage. Maybe this could work, if low magnitude of force were used, retracting the anterior segment and not being enough to move the posterior segment. Clinical studies have shown that there is no difference in anchorage loss between the two types of retraction.^36^

Normally, separate canine retraction is indicated to crowding cases or midline discrepancy cases. The mechanics (friction or frictionless) is practically the same. The orthodontist should bear in mind that using forces applied away from the center of resistance will result in tipping and rotation. Canine retraction can be carried out little enough to create space for incisors without flatting them. There is no reason to retract the anterior segment in two phases, unless crowding is present. Additionally, two phases can be unesthetic due to a more anterior gap, in addition to increasing treatment time. Another factor can be a more significant number of side effects, such as extrusion of incisors due to tipping back the canine crown, especially during sliding mechanics.^36^ ^,^ ^37^

In sliding mechanics, the orthodontist uses a guide wire. To retract canines, a stainless steel round-base archwire is used to slide canines distally. Normally, the canine is retracted with the use of 0.016-in and 0.018-in stainless steel wires in 0.018-in and 0.022-in slots, respectively. The reason is that the wire should be stiff enough for retraction, but should have deflection to fight against a potential tipping tendency. Rotation is another side effect that occurs as a result of sliding mechanics, and ligatures ties need to be used.^38^ ^,^ ^39^

## CONTROL OF MECHANICAL SIDE EFFECTS

Because the forces are not passing through the center of resistance, an additional moment should be provided when no rotation is necessary. A lingual attachment can be bonded, adding force on the lingual surface of the canine, so that the resultant force (buccal and lingual combined) passes through the center of resistance. Moreover, antirotation bends can be placed to prevent rotation during canine retraction by means of loops.^23^ ^,^ ^39^ ^,^ ^40^

After leveling canine retraction, side effects, such as reverse curve of Spee, can be generated. Uprighting of canines can produce mesial crown movement and create space between canines and premolars. Tie-back or power chain elastics can be used while uprighting of canines is performed. Also, a canine bypass is used to prevent side effects on adjacent teeth.

## EVALUATION OF SPACE CLOSURE AND CLINICAL CONSIDERATIONS

One of the most common problems after space closure is incisor torque. Round wire or undersized wire can lead to lingual tipping of the crown. Several methods are suggested to correct the undesired incisor torque, such as twisting the wire or using special springs.

Adding third order bends can be ineffective for several reasons. A full-size wire should be used to provide less play between wire and bracket. High-torque activation requires a very small amount of activation and frequent wire adjustment. Also, it is almost impossible to determine the amount of third-order bend providing enough M/F ratio. Adjacent bracket side effect can receive equal and opposite moments.

A torquing arch can be an alternative to produce ideal incisor torque. The force system produced by a torquing arch (i.e., 0.017 x 0.025-in TMA) results in adequate correction of incisor roots. A small amount of force as well as a high and continuous moment are produced because of the large arm. The incisors will receive the desired moment while undesired vertical force should be avoided using a stabilizing archwire. This system has the advantage of allowing easy visualization and measurement of torsional activations.^41^

In summary, there is no such thing as the best method of space closure. Some situations will require some techniques over others, and the orthodontist might have his or her own preferences. Regardless of the method to be used, a good understanding of biomechanics is essential. 
